# Supplementary material for: Inflammatory patterns in fixed airflow obstruction are dependent on the presence of asthma
Source: PLoS One. 2020 Dec 3;15(12):e0243109. doi: 10.1371/journal.pone.0243109 (PMC7714172; doi:10.1371/journal.pone.0243109)
Supplement: S1 Questionnaires — (DOCX) [file pone.0243109.s001.docx]

S1 Questionnaires

The questionnaires used in the NHANES data sets are published and available (accessed 2020-10-29):

2007-08:

Family Questionnaire, Smoking : <https://wwwn.cdc.gov/nchs/data/nhanes/2007-2008/questionnaires/smqfam07_08_eng.pdf>

Medical Conditions: <https://wwwn.cdc.gov/nchs/data/nhanes/2007-2008/questionnaires/mcq07_08_eng.pdf.pdf>

Respiratory Health: <https://wwwn.cdc.gov/nchs/data/nhanes/2007-2008/questionnaires/rdq07_08_eng.pdf>

Tobacco use: <https://wwwn.cdc.gov/nchs/data/nhanes/2007-2008/questionnaires/smq07_08_eng.pdf>

Audio Computer Assisted Personal Self Interview (ACASI) Questionnaire, Tobacco use: <https://wwwn.cdc.gov/nchs/data/nhanes/2007-2008/questionnaires/ai_smq_e.pdf>

Computer Assisted Personal Interview (CAPI), Tobacco use: <https://wwwn.cdc.gov/nchs/data/nhanes/2007-2008/questionnaires/mi_smq_e.pdf>

2009-2010

Family Questionnaire, Smoking: <https://wwwn.cdc.gov/nchs/data/nhanes/2009-2010/questionnaires/smqfam_f_eng.pdf>

Medical Conditions: <https://wwwn.cdc.gov/nchs/data/nhanes/2009-2010/questionnaires/mcq_f.pdf>

Respiratory Health: <https://wwwn.cdc.gov/nchs/data/nhanes/2009-2010/questionnaires/rdq_f.pdf>

Tobacco use: <https://wwwn.cdc.gov/nchs/data/nhanes/2009-2010/questionnaires/smq_f.pdf>

Audio Computer Assisted Personal Self Interview (ACASI) Questionnaire, Tobacco use: <https://wwwn.cdc.gov/nchs/data/nhanes/2009-2010/questionnaires/ai_smq_f.pdf>

Computer Assisted Personal Interview (CAPI), Tobacco use: <https://wwwn.cdc.gov/nchs/data/nhanes/2009-2010/questionnaires/mi_smq_f.pdf>

2011-2012

Family Questionnaire, Smoking: <https://wwwn.cdc.gov/nchs/data/nhanes/2011-2012/questionnaires/smq_family.pdf>

Medical Conditions: <https://wwwn.cdc.gov/nchs/data/nhanes/2011-2012/questionnaires/mcq.pdf>

Respiratory Health: <https://wwwn.cdc.gov/nchs/data/nhanes/2011-2012/questionnaires/rdq.pdf>

Tobacco use: <https://wwwn.cdc.gov/nchs/data/nhanes/2011-2012/questionnaires/smq.pdf>

Audio Computer Assisted Personal Self Interview (ACASI) Questionnaire, Tobacco use: <https://wwwn.cdc.gov/nchs/data/nhanes/2011-2012/questionnaires/smq_acasi.pdf>

Computer Assisted Personal Interview: <https://wwwn.cdc.gov/nchs/data/nhanes/2011-2012/questionnaires/smq_capi.pdf>
