# Supplementary material for: Inflammatory patterns in fixed airflow obstruction are dependent on the presence of asthma
Source: PLoS One. 2020 Dec 3;15(12):e0243109. doi: 10.1371/journal.pone.0243109 (PMC7714172; doi:10.1371/journal.pone.0243109)
Supplement: S1 Dataset — (DOCX) [file pone.0243109.s002.docx]

Datasets, above the link and below the name of the datasets used (available at the same web page).

2007-2008

Demographics data and sample weights

<https://wwwn.cdc.gov/nchs/nhanes/Search/DataPage.aspx?Component=Demographics&CycleBeginYear=2007>

Data file: Demographic Variables & Sample Weights

Examination data:

<https://wwwn.cdc.gov/nchs/nhanes/search/datapage.aspx?Component=Examination&CycleBeginYear=2007>

Data files: Body measures; Exhaled Nitric Oxide; Spirometry – Pre- and Post bronchodilator

Laboratory data: <https://wwwn.cdc.gov/nchs/nhanes/Search/DataPage.aspx?Component=Laboratory&CycleBeginYear=2007>

Data files: Complete Blood Count with 5-part Differential - Whole Blood; Cotinine - Serum & Total NNAL - Urine

Questionnaire data: <https://wwwn.cdc.gov/nchs/nhanes/Search/DataPage.aspx?Component=Questionnaire&CycleBeginYear=2007>

Data files: Medical Conditions: Respiratory Health, Prescription Medication; Smoking - Cigarette Use; Smoking - Household Smokers

2009-2010

Demographics data and sample weights

<https://wwwn.cdc.gov/nchs/nhanes/search/datapage.aspx?Component=Demographics&CycleBeginYear=2009>

Data file: Demographic Variables & Sample Weights

Examination data:

<https://wwwn.cdc.gov/nchs/nhanes/search/datapage.aspx?Component=Examination&CycleBeginYear=2009>

Data files: Body measures; Exhaled Nitric Oxide; Spirometry - Pre and Post-Bronchodilator

Laboratory data: <https://wwwn.cdc.gov/nchs/nhanes/search/datapage.aspx?Component=Laboratory&CycleBeginYear=2009>

Data files: Complete Blood Count with 5-part Differential - Whole Blood; Cotinine - Serum & Total NNAL - Urine

Questionnaire data: <https://wwwn.cdc.gov/nchs/nhanes/Search/DataPage.aspx?Component=Questionnaire&CycleBeginYear=2009>

Data files: Medical Conditions: Respiratory Health, Prescription Medication; Smoking - Cigarette Use; Smoking - Household Smokers

2011-2012

Demographics data and sample weights

<https://wwwn.cdc.gov/nchs/nhanes/search/datapage.aspx?Component=Demographics&CycleBeginYear=2011>

Data file: Demographic Variables & Sample Weights

Examination data:

<https://wwwn.cdc.gov/nchs/nhanes/search/datapage.aspx?Component=Examination&CycleBeginYear=2011>

Data files: Body measures; Exhaled Nitric Oxide; Spirometry - Pre and Post-Bronchodilator

Laboratory data: <https://wwwn.cdc.gov/nchs/nhanes/search/datapage.aspx?Component=Laboratory&CycleBeginYear=2011>

Data files: Complete Blood Count with 5-part Differential - Whole Blood; Cotinine - Serum & Total NNAL - Urine

Questionnaire data: <https://wwwn.cdc.gov/nchs/nhanes/search/datapage.aspx?Component=Questionnaire&CycleBeginYear=2011>

Data files: Medical Conditions: Respiratory Health, Prescription Medication; Smoking - Cigarette Use; Smoking - Household Smokers
